# Short-term creatine supplementation has no impact on upper-body anaerobic power in trained wrestlers

**DOI:** 10.1186/s12970-015-0107-6

**Published:** 2015-12-09

**Authors:** Martin Aedma, Saima Timpmann, Evelin Lätt, Vahur Ööpik

**Affiliations:** Institute of Exercise Biology and Physiotherapy, Estonian Centre of Behavioral and Health Sciences, University of Tartu, 18 Ülikooli St, Tartu, 50090 Estonia; Institute of Sport Pedagogy and Coaching, University of Tartu, 18 Ülikooli St, Tartu, 50090 Estonia

**Keywords:** Submission wrestling, Brazilian Jiu Jitsu, Ergogenic aid, Dietary supplement, Physical performance

## Abstract

**Background:**

Creatine (CR) is considered an effective nutritional supplement having ergogenic effects, which appears more pronounced in upper-body compared to lower-body exercise. Nevertheless, results regarding the impact of CR loading on repeated high-intensity arm-cranking exercise are scarce and in some cases conflicting. Interestingly, few of the conducted studies have structured their research designs to mimic real world sporting events. Therefore, our purpose was to address the hypothesis that CR ingestion would increase anaerobic power output in consecutive upper-body intermittent sprint performance (UBISP) tests designed to simulate wrestling matches on a competition-day.

**Methods:**

In a double-blind, placebo-controlled, parallel-group study, 20 trained wrestlers were assigned to either placebo or CR supplemented group (0.3 g ∙ kg^−1^ of body mass per day). Four 6-min UBISP tests interspersed with 30-min recovery periods were performed before (trial 1) and after 5 days (trial 2) of supplementation. Each test consisted of six 15-s periods of arm-cranking at maximal executable cadence against resistance of 0.04 kg ∙ kg^−1^ body mass interspersed with 40-s unloaded easy cranking periods and 5-s acceleration intervals (T1–T4). Mean power (MP), peak power (PP), fatigue index and heart rate parameters were measured during UBISP tests. Also, body weight and hydration status were assessed. Principle measures were statistical analysed with mixed-model ANOVAs.

**Results:**

Mean individual CR consumption in the CR group was 24.8 ± 2.5 g ∙ d^−1^. No significant (*P* > 0.05) differences occurred in body mass or hydration status indices between the groups or across trials. MP, PP and fatigue index responses were unaffected by supplementation; although, a significant reduction in MP and PP did occurred from T1 to T4 in both trial 1 and 2 (*P* < 0.001). Overall heart rate responses in the tests tended to be higher in the CR than PLC group (*P* < 0.05); but, trends in responses in trials and tests were comparable (*P* > 0.05).

**Conclusion:**

These results suggest that 5-day CR supplementation has no impact on upper-body muscle anaerobic power output in consecutive UBISP anaerobic tests mimicking wrestling matches on a competition day.

## Background

Creatine monohydrate is considered the most effective nutritional supplement in terms of increasing lean body mass and improving anaerobic performance [1]. Typical recommended creatine supplementation regimen consists of a loading phase of 20–25 g ∙ d^−1^ for 2–7 days, followed by a maintenance phase of 2–5 g ∙ d^−1^ thereafter [1–6]. A meta-analysis of 96 published investigations totaling 1847 subjects revealed that the effect of creatine supplementation is more pronounced in repetitive-bout than in single-bout exercise and in upper-body performance tasks than in lower body exercise [7]. Furthermore, the effect size for improvement in performance in high-intensity exercise lasting ≤ 30 s is greater than for tasks of longer duration, but no significant differences occur in performance effect between males and females or between trained athletes and untrained subjects [7].

In wrestling, repetitive forceful muscle contractions are required during most of sport’s maneuvers and the upper-body anaerobic power of an athlete is considered an important factor influencing competitive success [8, 9]. To this end, the findings of Branch [7] suggest that wrestlers may benefit from creatine supplementation, but surprisingly there are only a few previous studies which have examined the impact of creatine on physical performance indices in competitive wrestlers. Nevertheless, Koçak and Karli [10] demonstrated significant increases in mean power and peak power attained in a 30-s Wingate test after compared to before creatine supplementation in elite male Turkish wrestlers. Ziegenfuss et al. [11] studied elite male and female power athletes, including eight male wrestlers, and demonstrated significant improvement in repeated cycling sprint performance following short-term creatine supplementation. Ööpik et al. [12] found that creatine supplementation during 17-h recovery from rapid body mass loss stimulated the regain of physical working ability in a performance test simulating wrestling match in well-trained wrestlers.

The limitations in the data regarding the impact of creatine supplementation on physical performance in trained wrestlers are due to studies employing non-wrestling-specific test protocols [10, 11] or use extremely limited sample sizes (*n* = 5) [12]. A wrestling tournament usually lasts for many hours and in order to win a wrestler has to defeat on average four to five opponents. Whether creatine supplementation helps a wrestler to better maintain the ability to perform powerful actions throughout a tournament situation is not known.

Therefore, the aim of this study was to examine the influence of creatine monohydrate loading on upper-body intermittent sprint performance (UBISP) across four consecutive tests mimicking four wrestling matches on the same day. Considering the proved positive impact of creatine on performance in repeated high-intensity upper-body exercise in trained athletes [7], we hypothesized that creatine use compared to placebo would increase upper-body anaerobic power in trained wrestlers.

## Methods

### Subjects

Twenty healthy, male, amateur level Brazilian Jiu Jitsu and Submission Wrestling athletes participated in this study. The research protocol, which conforms to the ethical guidelines of the Declaration of Helsinki of the World Medical association, was approved by the Research Ethics Committee of the University of Tartu, Estonia (reference No 229/T-17). Written informed consent was obtained from the athletes prior to their participation in the study. All wrestlers trained at a local combative sports club and had a minimum of three years of regular wrestling training and two years of competition experience. During participation in the study, the athletes followed their usual training regimen. They did not participate in any competition or practiced any bodyweight control or cutting procedures. Their training volume was 6–8 h (3–4 2-hour sessions) per week. Each session included a combination of calisthenics exercises, technical and tactical drills, and training matches. Participants (mean ± SD) age, body mass, height and body fat percentage were 25.6 ± 3.8 years, 82.7 ± 8.6 kg, 185.1 ± 6.5 cm and 16.1 ± 2.4 %, respectively.

### Study design

The participants underwent two phases (preparatory and main) in this double-blind, placebo controlled study. In the preparatory phase, two visits, 2–3 days apart were made to the laboratory. During these visits the participants were familiarized with the testing devices and procedures and on both occasions a single familiarization UBISP test was performed. In the main phase of the study, the participants were divided into creatine (CR; *n* = 10) and placebo (PLC; *n* = 10) groups by listing their initial body mass from the lowest to the highest and then assigning them to alternate groups. There were no statistically significant differences between the groups for age, height, body mass or percentage of body fat. Both groups underwent two simulated competition days with exactly seven days in-between. The first simulated competition day (trial 1) was completed without any prior dietary supplementation. The second simulated competition day (trial 2) was completed after 5-day creatine monohydrate (CR group) or placebo (PLC group) supplementation period.

### Upper-body intermittent sprint performance test

The UBISP test protocol employed was a modification of that developed specifically for assessing upper body anaerobic performance in wrestlers [13]. The modified protocol was described in details by Aedma et al. [14] and it has been previously used for assessing the potential impact of different dietary supplements on physical performance in wrestlers [14, 15]. In brief, a cycle ergometer (Monark® Ergomedic 894 E, Monark, Sweden) with the pedals replaced by handgrips was employed. The UBISP test was 6-min, performed in a seated position and consisted of six separate 15-s bouts of arm-cranking exercise at maximal executable cadence against resistance of 0.04 kg · kg^−1^ body mass interspersed with 40-s unloaded easy cranking periods and 5-s acceleration intervals. Peak power (PP) and mean power (MP) for each 15-s bout of maximal effort was calculated by computer software (Monark, Sweden). Percent decrease in power (fatigue index) was calculated as following: [(PP—PP_bout6_)/PP] × 100.

### Simulated competition day

**Fig. 1:**
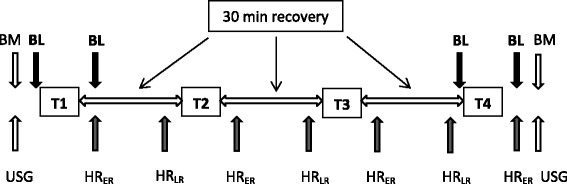
The sequence of procedures carried out during a simulated competition day. T1–T4—upper-body intermittent sprint performance tests; BM—body mass; BL—blood sample; USG—urine specific gravity; HR_ER_—early recovery heart rate; HR_LR_—late recovery hear rate

The participants were instructed to abstain from any training loads for 24 h prior to both trial 1 and trial 2. The participants reported to the laboratory after breakfast, approximately 9:00 in the morning. The sequence of procedures carried out on a trial day (i.e. simulated competition day) are shown in Fig. 1. Upon arrival, the participants provided a urine sample and their body mass was measured to the nearest 0.001 kg (CH3G-150I Combics scale, Sartorius, Germany). The participants then sat behind the cycle ergometer for 20 min. Five minutes prior to the first UBISP test of the day, they warmed up by performing a 3-min unloaded arm-cranking exercise at a self-selected pace. Then, after 2-min rest, participants accomplished four 6-min UBISP tests (hereinafter: T1, T2, T3 and T4) with 30-min recovery periods between the consecutive tests. During recovery, for the first 5 min following an UBISP test, the participants remained seated behind the ergometer. During the subsequent 10 min the participants could walk around in the laboratory, visit the toilet or rest in a self-selected position. In minutes 15–25 the participants rested quietly in a supine position on a couch. For the last 5 min of the recovery period the participants took a seat on the chair behind the ergometer and performed a 3-min warm-up by unloaded arm-cranking at a self-selected pace. Finally the participants remained seated and rested for the last 2 min of the recovery period. The ratings of perceived fatigue (RPF) and perceived exertion (RPE) were recorded immediately before and after each UBISP test, respectively. Both RPF and RPE in this study were measured using Borg’s 10-point scale [16]. After T2, during the 2nd recovery phase, the participants drank 0.5 L of sports drink Arctic Sport (A Le Coq, Estonia). During all the three recovery periods, the participants could drink water *ad libitum* and the individual quantities consumed were recorded. If they used the toilet, the participants were asked to collect all urine into a container.

### Creatine and placebo supplementation

The supplementation was initiated on the second day after trial 1 and lasted for five days prior to trial 2. During the supplementation period the CR group ingested creatine monohydrate (Creapure®, distributed by FAST Sports Nutrition, Func Food Finland OY, Finland) and the PLC group consumed glucose (Trauben Zucker, Müller’s Mühle GbmH, Germany). Both supplements were administered in gelatine capsules in a quantity of 0.3 g ∙ kg^−1^ of body mass per day. Daily dose of both supplements was divided into four equal portions and participants were instructed to consume these portions throughout a day, approximately 4 h apart. The creatine monohydrate supplementation protocol employed in the current study has been shown to induce significant increases in muscle total creatine content [17–19]. During the supplementation period the participants followed their routine training regimen and kept detailed food diary. Later analysis of these diaries revealed a 100 % compliance with ingesting of the supplements.

### Blood sampling and analyses

Altogether six fingertip capillary blood samples were drawn from each participant during both trial 1 and trial 2. To measure changes in plasma volume across the four consecutive UBISP tests, one sample was drawn immediately before T1 and another one after T4. Haemoglobin concentration and haematocrit were measured using a Celltac α MEK-6108 K (Nihon Kohden, Japan) blood analyser. The haemoglobin and haematocrit data were used for calculation of relative changes in plasma volume [20]. Blood lactate concentrations were measured in samples taken immediately before T1 and T4 (Pre-Ex) and 4 min after T1 and T4 (Post-Ex). Blood lactate concentration was measured using Dr. Lange cuvette tests LKM 140 and miniphotometer LP 20 Plus (Dr. Lange, Germany).

### Heart rate monitoring

Heart rate (HR) was monitored using standard Suunto HR belt (Suunto Oy, Finland) and recorded to a desktop computer using Suunto Monitor software (version 1.1.2 Suunto Oy, Finland). HR_PEAK_ was the highest HR value registered during UBISP test. Early recovery heart rate (HR_ER_) was recorded at the end of the 4th min of each recovery period. Late recovery heart rate (HR_LR_) was measured as the last 5 min average of the 10 min supine position resting phase during the recovery periods following T1, T2 and T3.

### Assessment of body composition and hydration status

Once following trial 1, before starting supplementation period, the participants underwent DXA screening (Lunar DPX IQ, Lunar Corp, USA) to determine their body composition.

Two urine samples for the assessment of hydration status were collected from the participants in both trial 1 and trial 2: before T1 and after T4. Urine specific gravity (USG) was measured using digital clinical refractometer PDX-CL (VEE GEE Scientific, Inc., Kirkland, WA). Additionally, the volume of urine passed was measured for the time interval from donation of the first urine sample till the end of the simulated competition day. Water retention was calculated as difference between the volumes of water consumed and urine passed.

### Statistical analysis

The Statistica™ version 12 software was used for statistical analysis. All data are expressed as mean ± SD. Normality of all data sets was examined using the Kolmogorov-Smirnov test. The effect of creatine ingestion on performance (PP, MP, fatigue index), heart rate (HR_PEAK_, HR_ER_, HR_LR_), RPE and RPF was assessed using 3-way mixed model ANOVA (between-factor—group, within factors—trial and test). The blood lactate data were analyzed with 4-way mixed model ANOVA (between-factor—group, within factors—trial, test, and pre- and post-performance test). Body mass data were analyzed using 3-way mixed model ANOVA (between-factor—group, within factors—trial and time). Hydration data were analyzed using 2-way mixed model ANOVA (between factor—group, within factor—trial). The nutritional data were analyzed using Student’s *t*-test for independent variables. In all analyses, where a significant effect was observed, a Tukey’s HSD post-hoc was used to identify specific differences. Statistical significance was set at *P* < 0.05.

## Results

### Energy, macronutrients and water consumption

During the 5-day supplementation period the self-reported daily energy intake of the participants was 2703 ± 780 kcal in PLC group and 2362 ± 454 kcal in CR group. In PLC group, the total daily energy intake was made up of carbohydrates, proteins and fats in proportions of 38.7 ± 13.5 %, 19.9 ± 6.4 %, and 41.5 ± 11.3 %, respectively. In CR group carbohydrates, proteins and fats contributed to the total energy intake by 35.6 ± 7.4 %, 18.1 ± 3.1 % and 45.6 ± 7.0 %, respectively. The self-reported daily water consumption was 2481 ± 906 ml in PLC group and 2250 ± 1003 ml in CR group. There were no statistically significant between-group differences in any of the nutritional parameters assessed (*P* > 0.05). Mean daily CR consumption of the participants in the CR group was 24.8 ± 2.5 g.

### Body mass and hydration status

Body mass and hydration status indices are presented in Tables 1 and 2, respectively. There were no significant (*P* > 0.05) differences in body mass between the supplementation groups, within the two trials, or in relation to before T1 and after T4.

**Table 1:** Body mass (kg)

	Placebo (*n* = 10)		Creatine (*n* = 10)	
	Trial 1	Trial 2	Trial 1	Trial 2
Before T1	82.64 (9.36)	82.49 (9.31)	82.73 (8.38)	82.46 (8.65)
After T4	82.87 (9.36)	82.66 (9.41)	82.76 (8.13)	82.53 (8.38)
Change	0.23 (0.43)	0.17 (0.32)	0.03 (0.52)	0.06 (0.58)

Data are presented as mean (SD). T1 and T4—upper-body intermittent sprint performance tests

**Table 2:** Hydration indices

Parameter	Placebo (*n* = 10)		Creatine (*n* = 10)	
	Trial 1	Trial 2	Trial 1	Trial 2
USG before T1	1.0220 (0.0061)	1.0194 (0.0084)	1.0167 (0.0069)	1.0216 (0.0075)
USG after T4	1.0197 (0.0048)	1.0184 (0.0041)	1.0184 (0.0029)	1.0192 (0.0036)
Urine volume (ml)	194 (95)	240 (100)	281 (141)	291 (250)
Water intake (ml)	1033 (427)	955 (322)	1003 (418)	1047 (428)
Water retention (ml)	839 (431)	716 (341)	723 (431)	757 (439)
Change in PV (%)				
Before T1 → After T4	−2.69 (5.13)	−3.59 (5.69)	−4.63 (4.80)	−1.82 (4.80)

Data are presented as mean (SD). Urine volume, water intake and water retention were measured for the time interval between before T1 and after T4. *USG* urine specific gravity; *PV* plasma volume; T1 and T4—upper- body intermittent sprint performance tests

All hydration indices USG, urine volume, water intake and water retention display similar responses to body mass. That is, there were no significant (*P* > 0.05) differences in hydration indices between the supplementation groups, within the two trials, or in relation to tests (i.e. USG before T1 and after T4).

Finally, calculated plasma volume shifts did not differ significantly (*P* > 0.05) between supplement groups or across trials.

### Upper body intermittent sprint performance and fatigue index

**Fig. 2:**
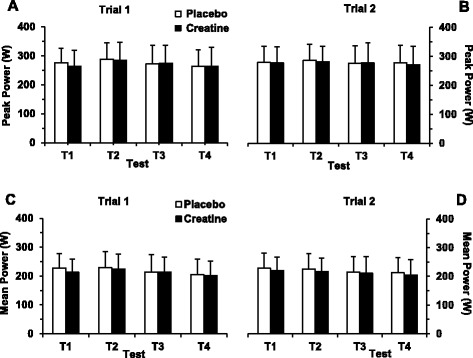
Peak power (**a**, **b**) and mean power (**c**, **d**) attained during upper-body intermittent sprint performance tests T1–T4 before (trial 1) and after (trial 2) 5-day supplementation period. The columns represents average ± SD peak and mean power values for six 15-s bouts of maximal effort performed during the test. In both placebo and creatine groups the number of subjects is 10

Figure 2 depicts the changes in PP and MP for each group in both trials and across all tests (T1–T4). The only significant finding in this data was that for both PP and MP there was a significant reduction (*P* = 0.001 and *P* < 0.001, respectively) due to exercise (main effect) from T1 to T4. No effects due to supplement group or trial were detected. In both CR and PLC groups, six participants attained greater and four participants smaller PP (average for four tests) after compared to before supplementation period. The ranges of individual improvements in average PP in CR and PLC groups were 3.3–20.9 W and 6.2–20.6 W, respectively. The ranges of individual decrements in average PP in CR and PLC groups were 2.2–10.7 W and 2.8–10.5 W, respectively. Regarding MP (average for four tests), five participants in CR group and six participants in PLC group revealed improvements after compared to before supplementation period in the ranges of 1.0–26.4 W and 1.2–23.1 W, respectively. Average MP decreased in five participants in CR group (range 1.7–24.0 W) and in four participants in PLC group (range 1.5–15.1 W). Fatigue index responses displayed no significant (*P* > 0.05) changes (data not reported).

### Blood lactate concentration

**Fig. 3:**
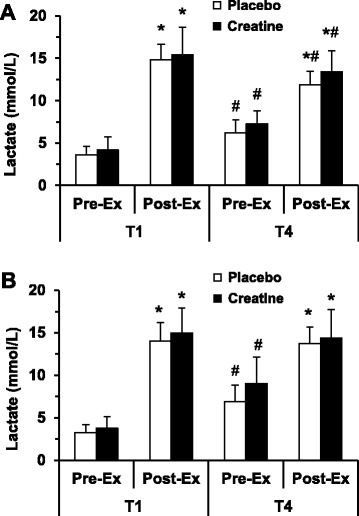
Blood lactate in upper-body intermittent sprint performance (UBISP) tests T1 and T4 before (**a**) and after (**b**) 5-day supplementation period. Data are presented as means ± SD for 10 subjects in both placebo and creatine groups. Pre-Ex—immediately before UBISP test; Post-Ex—4 min after UBISP test. Significantly different (*P* < 0.05): * from Pre-Ex: ^#^ from T1

Lactate responses are displayed in Fig. 3. Post-exercise significant increases in lactate occurred in T1 and T4 for both the PLC and CR groups (i.e., main effect). Additionally, the Pre-Ex lactate concentrations at T4 for trial 1 and trial 2 were significantly greater than in T1 (*P* values range: 0.0002–0.003).

### Heart rate

All forms of HR response measurements are reported in Table 3. The CR group had significantly greater overall HR_PEAK_ (*P* = 0.017) and HR_ER_ (*P* = 0.043) response compared to PLC group. Furthermore, for tests the HR_ER_ and HR_LR_ significantly decreased overall (*P* values 0.00002 and 0.03, respectively).

**Table 3:** Heart rate (bpm)

Test	Placebo (*n* = 10)			Creatine (*n* = 10)		
	HR_PEAK_	HR_ER_	HR_LR_	HR_PEAK_	HR_ER_	HR_LR_
Trial 1						
T1	165 (9)	105 (11)	80 (10)	172 (11)	115 (15)	87 (13)
T2	164 (7)	104 (11)	80 (8)	172 (8)	114 (15)	86 (13)
T3	165 (7)	101 (9)	76 (9)	172 (6)	110 (15)	85 (12)
T4	165 (6)	98 (9) ^a^	―	174 (7)	106 (15) ^a^	―
Trial 2						
T1	163 (8)	101 (8)	78 (10)	171 (9)	115 (15)	85 (14)
T2	164 (5)	102 (9)	77 (7)	172 (9)	115 (14)	85 (15)
T3	162 (5)	100 (8)	74 (8)	171 (8)	111 (13)	84 (14)
T4	165 (6)	100 (8)	―	173 (8)	112 (12)	―

Data are presented as mean (SD). *HR*_*PEAK*_ peak heart rate; *HR*_*ER*_ early recovery heart rate; *HR*_*LR*_ late recovery heart rate; T1–T4—upper-body intermittent sprint performance tests

^a^ Different from T1, *P* < 0.05

### Ratings of perceived fatigue and exertion

The RPF and RPE results are reported in Table 4. For RPF, in the PLC group a significant (*P* < 0.001) increase fatigue occurred in trial 1 and 2. In trial 1 T2, T3 and T4 were significantly greater than T1; and, in trial 2 T3 and T4 were significantly greater than T1. In the CR group the only significant change in RPF was in trial 2 where T4 was greater than T1.

**Table 4:** Ratings of perceived fatigue (RPF) and perceived exertion (RPE)

	Test	Placebo (*n* = 10)		Creatine (*n* = 10)	
		Trial 1	Trial 2	Trial 1	Trial 2
RPF	T1	1.1 (0.8)	0.9 (0.7)	1.8 (0.9)	1.3 (1.1)
	T2	1.9 (1.1) ^a^	1.9 (1.2)	2.2 (0.8)	1.8 (1.1)
	T3	2.0 (1.3) ^a^	1.9 (1.0) ^a^	2.3 (0.8)	1.9 (1.1)
	T4	2.0 (1.0) ^a^	2.3 (0.9) ^a^	2.5 (1.2)	2.3 (1.4) ^a^
RPE	T1	6.8 (2.1)	6.9 (1.7)	5.9 (1.7)	5.8 (1.7)
	T2	7.2 (1.7)	7.6 (1.6)	6.3 (1.4)	6.2 (1.5)
	T3	7.6 (1.7)	7.5 (1.5)	6.5 (1.4)	6.3 (1.6)
	T4	8.1 (1.3) ^a^	7.8 (1.2)	7.1 (1.5) ^a^	6.6 (1.7)

Data are presented as mean (SD). T1–T4—upper-body intermittent sprint performance tests. ^a^ Different from T1, *P* < 0.05

The only significant (*P* < 0.05) change in RPE occurred in trial 1 for both the PLC and CR groups. The T4 responses in both of these situations were greater than T1 showing an increase in effort.

## Discussion

The main finding of this study was that short-term creatine supplementation had no impact on PP or MP attained by trained wrestlers during consecutive anaerobic tests intended to mimic a typical competition-day in wrestling. Furthermore, creatine did not influence fatigue development substantially relative to the placebo. To the best of our knowledge, this is the first study assessing the potential efficacy of creatine loading in improving upper-body anaerobic performance in combative-type sports under a simulated competition setting. Previous studies focusing on wrestling scenarios have demonstrated ergogenic effects of creatine supplementation in participants, but as noted earlier these studies have limitations [10–12].

Our hypothesis that creatine loading would increase upper-body anaerobic power was based on the outcomes of meta-analytic and systematic literature reviews. These reviews revealed that creatine supplementation has the most pronounced positive effect on performance in multiple bouts of high-intensity, short-duration exercise [2, 4, 7, 21, 22] and that generally the effect of creatine on performance is greater in upper-body exercise than in lower-body exercise [7]. Regarding intermittent sprint performance in exercise tasks loading lower-body musculature, marked improvement following short-term creatine supplementation has been reported by numerous research groups in untrained and moderately trained subjects [23–29] as well as in highly trained athletes [11, 30–35]. Not all of the relevant studies, however, have confirmed the ergogenic effect of creatine loading [36–40]. Furthermore, to the best of our knowledge, only two research groups [41, 42] have assessed the potential impact of short-term creatine supplementation on repeated high-intensity arm-cranking exercise performance in athletes and the findings of these studies were conflicting. Grindstaff et al. [41] reported 7.5 % greater peak power output during the first of three consecutive 20-s arm ergometer maximal-effort sprint tests after compared to before nine days of creatine monohydrate (21 g ∙ d^−1^) ingestion in trained swimmers. Green et al., however, found that six days of creatine supplementation (20 g ∙ d^−1^) did not enhance peak power or mean power during three consecutive 30-s arm Wingate tests in recreational athletes [42]. In addition, Jacobs et al. [43], employing incremental peak arm ergometry test consisting of 2-min work stages and 1-min recovery periods, observed a significant increase in peak power output (6.7 %) following seven days of creatine supplementation (20 g · day^−1^) in men with complete cervical-level spinal cord injury.

Taken together, the limited and inconsistent literature and the current findings are clearly insufficient to draw any definitive conclusions regarding the influence of short-term creatine supplementation on anaerobic performance in arm-cranking exercise. Therefore, further studies are warranted in order to better elucidate the potential impact of creatine on performance in this particular mode of physical activity.

In our participants, creatine loading had no influence on body mass. According to Branch et al. [7], approximately 64 % of studies which have measured body mass and/or body composition have reported statistically significant increases in body mass or lean body mass due to creatine supplementation. Regarding short-term creatine monohydrate loading in particular, a review conducted by Volek and Kraemer [44] revealed that ingestion of 20–30 g of this substance per day for five to seven days usually induces an increase in body mass in the range of 0.9–1.8 kg. Kilduff et al. [45] reported positive correlation between change in body mass and whole-body creatine uptake. Saab et al. [46] suggested that an increase in intracellular muscle water may occur due to osmotic properties of creatine and this may be responsible for the rapid change in body mass accompanying creatine use. Therefore, some authors [28, 34] have interpreted increase in body mass as an indirect evidence of successful muscle creatine loading.

The absence of any change in body mass or hydration in our subjects during the supplementation period does not necessarily, however, indicate that creatine ingestion failed to increase muscle creatine content. Some reports indicate body mass actually does not correlate with increases in muscle creatine content [18] or in the muscle phosphocreatine/ATP ratio [46]. Furthermore, short-term creatine supplementation has been shown to improve performance in high-intensity arm-cranking exercise [41], cycling sprints [25, 27, 29], maximal unilateral knee extension and maximal deadlift strength [47] and to increase anaerobic running capacity [48] without concomitant changes in body mass.

Creatine ingestion did not influence blood lactate response to the anaerobic tests in our participants. The potential effect of creatine on blood lactate levels in repeated high-intensity arm-cranking exercise has not been reported, but blood lactate response to repeated running or cycling sprints have been shown to be unchanged [25, 34], increased [26, 35], or decreased [23, 31] when compared to before creatine loading.

HR_PEAK_ and HR_ER_ were consistently higher in CR than PLC group. The reason for this finding is unclear, but the data clearly reveal that supplements ingested (creatine and placebo) had no impact on HR during the UBISP tests or during recovery periods between the consecutive UBISP tests in our participants. Regarding HR_PEAK_, our findings are consistent with that reported by Jacobs et al. [43], who observed similar peak HR values before and after creatine loading in cervical cord injury patients performing incremental peak arm ergometry test. Others have reported no effect of creatine on exercise heart rate in repeated running [32, 33] or cycling [27] sprints or lower average heart rate during repeated running sprints after compared to before creatine loading [31], or during recovery [27].

There was no substantial and consistent effect of creatine or placebo supplementation on the RPF or RPE responses to the UBISP tests. This finding suggests that creatine loading did not strongly influence perception of effort during the tests or subjective feeling of recovery between the consecutive tests. Jacobs et al. examined RPE during arm-cranking exercise and reported similar RPE values in creatine and placebo exercise trials [43]. However, as significantly greater peak power output was attained after creatine loading compared to placebo, the RPE data [43] suggest that creatine actually attenuated the perception of effort in their subjects.

An acknowledged methodological limitation of this study is the absence of direct evidence confirming that creatine loading increased muscle creatine content in our subjects. Considering that the extent of creatine uptake during supplementation period appears to be critical to subsequent performance in repeated bouts of high-intensity exercise [17, 19], failure to increase muscle creatine content would explain the lack of any change in upper body anaerobic power. However, we are confident in the high quality of the creatine product and our subjects complied with supplement use based upon food diary reviews and in direct questioning of them individually. The potential influence of participation in routine wrestling training sessions on muscle creatine content in our subjects is difficult to evaluate, because exercise has been demonstrated to increase [49, 50], have no impact [51] or decrease [52] muscle creatine accumulation during loading period. Nevertheless, we cannot completely exclude the possibility that a longer supplementation period might have been needed for occurrence of an ergogenic effect. Vandenberghe et al. [53] and Francaux et al. [54] administered similar daily dosages of creatine (20 g and 21 g) to their subjects for 4 or 14 days, respectively. While both groups reported significant increases in muscle phosphocreatine content following supplementation period, the extent of the increase was much smaller (approximately 5 %) after 4 days [53] compared to that observed after 14 days (approximately 20 %) [54].

The exercise protocol employed was developed to be in line with a “real world” situation for mimicking wrestling matches during a competition-day and hence practical. On the other hand, the specificity of the exercise protocol may have diminished the potential ergogenic effect of increased muscle creatine content in our subjects. Improved performance in repeated bouts of high-intensity exercise following creatine loading is likely due to an increased pre-exercise creatine phosphate availability and enhanced resynthesis of phosphocreatine during recovery periods [17, 23, 24]. However, it has been demonstrated that the rate of resynthesis of phosphocreatine is practically identical in creatine-loaded and non-loaded muscle during the initial 40 s after intense exercise, whereas the accelerated resynthesis mainly occurs during the 2nd minute of recovery [18]. While phosphocreatine resynthesis depends on muscle oxygen supply and removal of lactate and protons [55], i.e. processes which largely are based on diffusion [56], the 40-s unloaded easy cranking periods (active recovery intervals) between consecutive 15-s maximal exercise bouts may be too short to elicit enhanced phosphocreatine resynthesis in creatine-loaded state. Further, high-intensity upper-body exercise relies much more on the anaerobic lactic energy system than lower-body exercise [57, 58], but the diffusional area is smaller and the diffusional distance longer in the arm than in the leg muscles [59]. Therefore, occurrence of the potential ergogenic effect of creatine loading in repeated bouts of high-intensity arm-cranking exercise may require longer between-bout recovery intervals than those used in the current study or in the other relevant studies [41, 42].

## Conclusion

Five days of creatine monohydrate supplementation failed to increase upper-body anaerobic power in anaerobic tests mimicking wrestling matches in trained wrestlers. Considering the great practical importance of upper body muscular performance not only for combat sports athletes but also for disabled people who use wheelchair in their everyday life, further studies in this area of interest are warranted.

## Abbreviations

CR: Creatine
HR: Heart rate
MP: Mean power
PLC: Placebo
Post-Ex: After exercise
PP: Peak power
Pre-Ex: Before exercise
RPE: Rating of perceived exertion
RPF: Rating of perceived fatigue
UBISP: Upper-body intermittent sprint performance
USG: Urine specific gravity
